# An L-band interferometric synthetic aperture radar study on the Ganos section of the north Anatolian fault zone between 2007 and 2011: Evidence for along strike segmentation and creep in a shallow fault patch

**DOI:** 10.1371/journal.pone.0185422

**Published:** 2017-09-29

**Authors:** Marcello de Michele, Semih Ergintav, Hideo Aochi, Daniel Raucoules

**Affiliations:** 1 Natural Risks department, French Geological Survey (BRGM), Orléans, France; 2 Department of Geodesy, Boğaziçi University, Kandilli Observatory and Earthquake Research Institute, Istanbul, Turkey; Gaziosmanpasa Universitesi, TURKEY

## Abstract

We utilize L-band interferometric synthetic aperture radar (InSAR) data in this study to retrieve a ground velocity map for the near field of the Ganos section of the north Anatolian fault (NAF) zone. The segmentation and creep distribution of this section, which last ruptured in 1912 to generate a moment magnitude (Mw)7.3 earthquake, remains incompletely understood. Because InSAR processing removes the mean orbital plane, we do not investigate large scale displacements due to regional tectonics in this study as these can be determined using global positioning system (GPS) data, instead concentrating on the close-to-the-fault displacement field. Our aim is to determine whether, or not, it is possible to retrieve robust near field velocity maps from stacking L-band interferograms, combining both single and dual polarization SAR data. In addition, we discuss whether a crustal velocity map can be used to complement GPS observations in an attempt to discriminate the present-day surface displacement of the Ganos fault (GF) across multiple segments. Finally, we characterize the spatial distribution of creep on shallow patches along multiple along-strike segments at shallow depths. Our results suggest the presence of fault segmentation along strike as well as creep on the shallow part of the fault (i.e. the existence of a shallow creeping patch) or the presence of a smoother section on the fault plane. Data imply a heterogeneous fault plane with more complex mechanics than previously thought. Because this study improves our knowledge of the mechanisms underlying the GF, our results have implications for local seismic hazard assessment.

## Introduction

### Tectonic context of the study area

**Fig 1:**
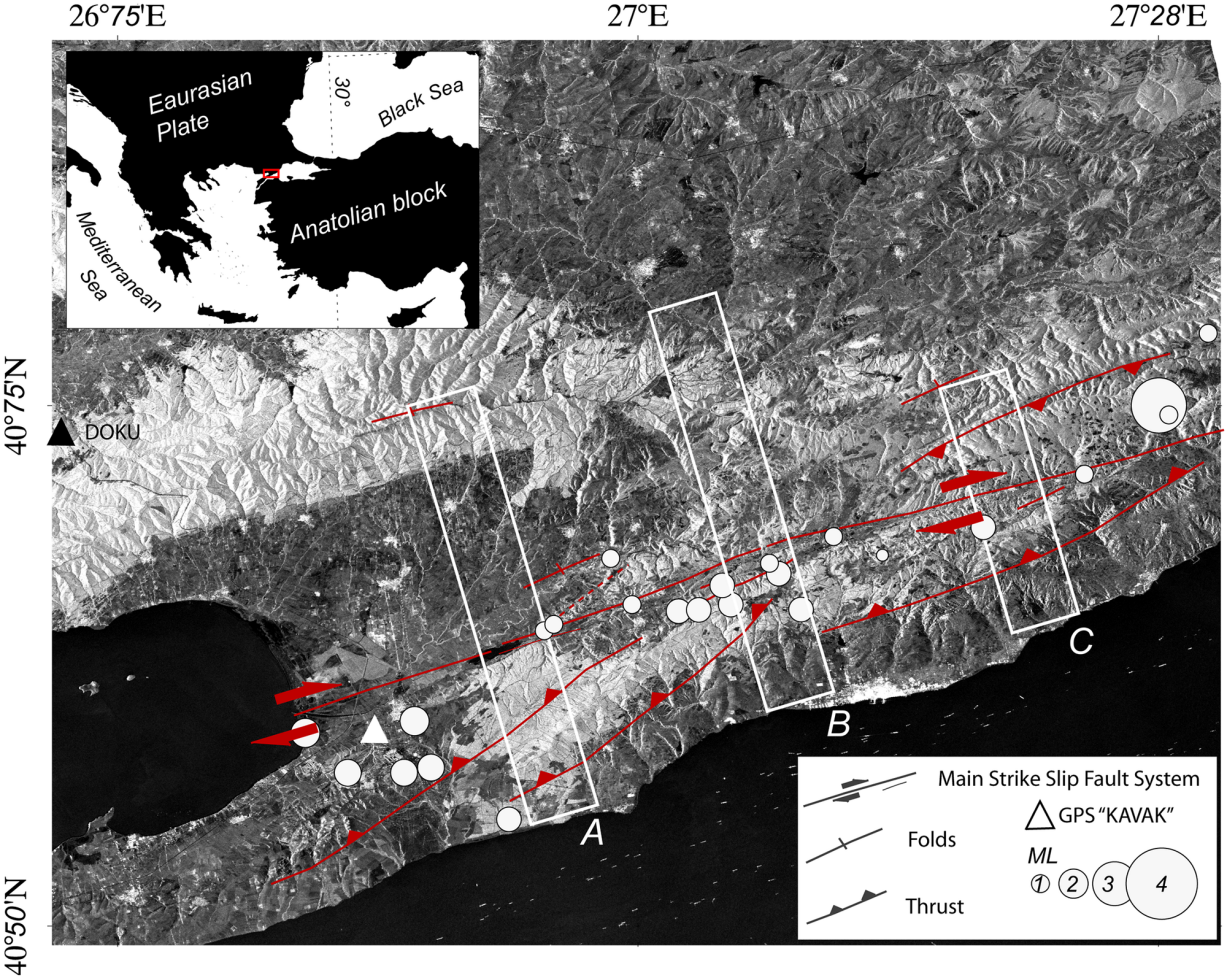
The study area. Map of the study area discussed in this paper. The background image is the mean SAR amplitude image, calculated from ALOS PALSAR data. Simplified fault traces from Yaltirak and Alpar (2002) are shown in red; white circles represent earthquakes (Koeri catalogue); white boxes denotes areas used for profile stacking A, B, C. Red arrows indicate the sense of fault motion. Triangles represent GPS locations: black triangle is DOKU, white triangle is KAVAK.

The north Anatolian fault (NAF) is a major right-l ateral strike-slip fault with a length of about 1,500 km and a roughly east-west strike. This fault is thought to be the tectonic boundary between the Anatolian and Eurasian plates in northern Turkey (e.g. [1–3]). Geologic, geodetic, and seismologic evidence have been used to demonstrate that the NAF accommodates between ca. 14 mm/yr and ca. 30 mm/yr of relative plate motion (e.g. [4–6]); the westernmost section of the NAF, the Gazikoy-Saros segment (also called the Ganos fault, GF), is the onshore section of the northern strand of the NAF (e.g. [7]). The strike of the GF runs between the Sea of Marmara and the Gulf of Saros (Fig 1), while the Ganos section of the NAF remains seismologically active. This section last ruptured in 1912 generating a Mw7.3 earthquake that fractured the entire inland fault segment to a length of about 50 km. Field observations show that this earthquake produced a right-lateral strike-slip offset of at least 3 m (e.g. [8, 9]). A review of the literature reveals additional historical reports of other large earthquakes that can be attributed to the GF that occurred in A.D. 824, A.D. 1343, A.D. 1509, and A.D. 1766 (e.g.[10–12]). The GF is believed to link the northern strand of the NAF zone in the Sea of Marmara with the north Aegean trough where slip partitioning results in branching of the fault zone (e.g. [13, 14]).

According to Okay et al. [14], the GF consists of several sub-parallel faults that share a dominant right-lateral strike-slip motion that was initiated towards the end of the Miocene leading to transpressional uplift that formed the Ganos Mountains. Geodetic studies suggest that present-day normal fault convergence is accommodated by the GF at a rate of 1.1 ± 0.4 mm/yr (e.g. [15]), while Tuysuz et al. [3] interpreted the apparent lack of seismicity of Mw larger than 3 along this fault as indicative of a locked segment that only slips during large earthquakes. Studies based on the inversion of global positioning system (GPS) time series and C-band interferometric synthetic aperture radar (InSAR) data have suggested that this section of the NAF creeps at a depth between 8 km and 17 km and as a single segment at a rate of 2 cm/yr (e.g. [7, 15]). At the same time, geological field investigations have led to interpretation of the Ganos section as the result of multiple faults segments [7]. Nevertheless, it remains unclear just how present-day strain is accumulated along the GF and whether, or not, the Ganos section is segmented at shallow depths. Both past and recent studies based on InSAR have proved successful in measuring surface fault motion on the NAF, particularly at the Ismetpasa creeping section (e. g. [4, 16–18]).

In this study, we process L-band InSAR data from the ALOS Phased Array type L-band SAR (PALSAR) sensor that belongs to the Japanese Aerospace Exploration Agency (JAXA) that encompasses the Ganos section of the NAF. Although the ALOS PALSAR archive for this study area is not densely populated if just a single polarization acquisition mode is considered, a much larger number of SAR scenes are available if both single and dual polarization acquisition modes are taken into account. We therefore interferometrically combined both single and dual polarization acquisition modes in this study to ensure a sufficiently populated archive to allow stacking. This study has three main aims. First, we investigate whether, or not, it is possible to retrieve ground velocity maps for this study area from the stacking of L-band interferograms, combining both single and dual polarization SAR acquisition modes. Second, we determine whether a ground velocity map can be used to complement GPS observations to differentiate present-day surface displacement in the near field of multiple segments of the GF fault. Third, we discuss whether, or not, it is possible to characterize the spatial distribution of shallow creep on multiple segments at shallow depths. To accomplish these aims, we first calculated all possible interferograms for the period between 2007 and 2011 coupling single and dual polarization ALOS PALSAR data, unwrapped and stacked the most coherent, and interpreted our results using a simple elastic dislocation model.

### Data acquisition and processing

InSAR data can be used to map ground deformation at a spatial resolution of tens of meters with sub-centimeter precision along the satellite line-of-sight direction (LOS) [19–21]. Previous work has shown that when dealing with interseismic strain measurements, L-band InSAR (wavelength of 23,5 cm) performs better than shorter wavelength SAR for vegetated areas, such as the region in this study (e.g. [22]). Ascending orbit satellite-path orientation with respect to fault orientation is optimal in order to obtain suitable InSAR LOS sensitivity relative to strike-slip movement, parallel to the NAF in the Ganos section. Besides, for InSAR process sing to succeed, SAR scenes must be acquired at the same radar band, and with the same LOS angle, and polarization. One major disadvantage of the ALOS PALSAR archive for the region of the Ganos section of the NAF is that data are not particularly abundant in terms of the number of SAR scenes that encompass the same viewing angle and polarization mode. Thus, we enhanced the number of potential scenes covering the GF by joint processing of multiple polarization data, acquiring both Fine Beam Single mode (FBS) and Fine Beam Dual mode polarization (FBD) PALSAR data with LOS angles of 38.7°. Of these, the FBD data matrix consists of data columns that alternately record single polarizations, HH + HV respectively, while the FBS data matrix just comprises HH polarization signals, where H stands for Horizontal polarization and V stands for Vertical polarization. The drawback of this approach is that we are only able to process these data at the same time at the cost of a decrease in spatial resolution, while improving temporal sampling and dataset size. In practice, this can be achieved by extracting one data column out of every two from both the HH FBS and HH-HV FBD data matrices. This generates a series of HH FBS and HH FBD data that has the same polarization but a 50% reduced range of spatial resolution (half of the original resolution). This reduction will not, however, interfere with the results of this study as we are not interested in metric scale phenomena. We implemented this processing step using GAMMA routines [23], resulting in a final 14 single look complex (SLC) L-band dataset that spans four years, from July 7^th^, 2007, to January 15^th^, 2011, with a LOS resolution of 20m (Table 1).

**Table 1:** ALOS PALSAR dataset.

date	Mode	orbit	Look angle
07/07/2007	FBS	ascending	38,7°
07/10/2007	FBS	ascending	38,7°
08/04/2008	FBD	ascending	38,7°
24/05/2008	FBS	ascending	38,7°
24/08/2008	FBS	ascending	38,7°
09/10/2008	FBS	ascending	38,7°
24/02/2009	FBD	ascending	38,7°
12/07/2009	FBS	ascending	38,7°
27/08/2009	FBS	ascending	38,7°
12/10/2009	FBS	ascending	38,7°
30/05/2010	FBS	ascending	38,7°
15/07/2010	FBS	ascending	38,7°
30/08/2010	FBS	ascending	38,7°
15/01/2011	FBD	ascending	38,7°

The table shows the ALOS PALSAR dataset available on the study area. It includes both FBS and FBD polarization modes, ascending orbit and a look angle of 38,7°.

Atmospheric delays can mask weak shallow creep signals in the case of single interferograms [24, 25]. Therefore, to reduce the extent of the atmospheric influence on interferometric phase, we used a stacking methodology. Moreover, our aim was not to detect temporal variations in fault motion as we assumed that tectonic-related deformation rates remained constant over the observation period. We therefore employed the stacking method implemented in the software GAMMA [23], on a set of selected interferograms, starting from 14 RAW ALOS PALSAR images that we focused to obtain 14 SLCs. Because L band SAR data are heavily affected by radio frequency interference (RFI) across the area, which leads to co-registration problems and or interferogram streaks, we carefully filtered this effect during the focusing procedure, following the procedure described in [26]. Then, we co-registered the 14 SLCs on the basis of a common master image (the first acquisition) and we calculated all possible interferograms using a multi looking of 1 (LOS) and 5 (azimuth). This procedure yielded 91 multilooked interferograms.

Topographic contributions to interferometric phase were calculated for each interferogram using the Shuttle Radar Topography Mission 90 m digital elevation model (DEM) and were subtracted from the interferograms [27]. Then, on the basis of 91 initial differential interferograms, we selected a subset of 39 high-signal-coherence examples via visual analysis (Table 2). We chose interferograms with a mean signal coherence of greater than 0.6 for at least 75% of each interferogram. Then, we applied the Minimum Cost Flow (MCF) algorithm [28], again implemented in the software GAMMA, to unwrap selected interferograms. In each case, the unwrapping step, performed at the original resolution of the grid, was improved using a phase reference model obtained by unwrapping corresponding multiple- look interferograms as these are simpler to process. We then resized the phase reference model to the original pixel resolution of the resolution grid, while an unwrapped phase value for each pixel was computed using the interferogram of complex values and the assumption that phase values in the resized model will correspond to the correct unwrapped phase within the interval ± π. Thus, the resulting unwrapped phase fulfils the condition that re-wrapping of the unwrapped phase will result in exactly the phase of the complex interferogram, to the exclusion of a constant offset which can be defined via the phase indicated for the reference location [29].

**Table 2:** The ALOS PALSAR interferograms used for stacking.

date (Master)	date (Slave)	time spam (days)	Baseline ┴ (m)
20071007	20080408	184	184
20071007	20090224	506	506
20071007	20090712	644	644
20071007	20090827	690	690
20071007	20091012	736	736
20080408	20090224	322	322
20080408	20090712	460	460
20080408	20090827	506	506
20080408	20091012	552	552
20080408	20100530	782	782
20080824	20081009	46	46
20080824	20090224	184	184
20080824	20090712	322	322
20080824	20091012	414	414
20081009	20090224	138	138
20081009	20090712	276	276
20081009	20091012	368	368
20090224	20090712	138	138
20090224	20090827	184	184
20090224	20091012	230	230
20090224	20100830	552	552
20090712	20090827	46	46
20090712	20091012	92	92
20090712	20100530	322	322
20090712	20100715	368	368
20090712	20100830	414	414
20090712	20110115	552	552
20090827	20091012	46	46
20090827	20100530	276	276
20090827	20100830	368	368
20091012	20100530	230	230
20091012	20100715	276	276
20091012	20100830	322	322
20091012	20110115	460	460
20100530	20100715	46	46
20100530	20100830	92	92
20100530	20110115	230	230
20100715	20100830	46	46
20100715	20110115	184	184

The 39 interferograms that were used to build the stacks reported in this paper, including dates, time spans, and perpendicular baseline (less than 800 m).

We also estimated altitude-related atmospheric phase delay. Depending on atmospheric conditions, path delay can be dependent on altitude as the result of variations in atmospheric water vapor and pressure profiles between the acquisition times of interferometric image pairs (e.g. [30]). Thus, to mitigate this, we used the software GAMMA to determine the linear regression coefficients of the residual phase with respect to height in unwrapped interferograms, as well as a DEM projected in the same geometry as the sensor to generate a phase model of height-dependent atmospheric phase delay for each unwrapped interferogram. Each model was then subtracted from the corresponding unwrapped single interferogram, and a stacking algorithm was used to estimate the linear rate of differential phase, via the set of unwrapped differential interferograms, to derive a time-averaged linear velocity map for the study area. This stacking algorithm uses individual interferogram phases divided by time interval weighted by the square of each to estimate phase rate, as proposed by Le Mouelic et al. [31]. The underlying assumption of this approach is that atmospheric conditions are not stationary across the set of interferograms. Finally, because this process calculates a phase ramp on the full PALSAR frame and removes it form the final stack, long wavelength tectonic motion in the far-field generated by the movement of faults at depth is invisible in the velocity field retrieved by InSAR. Nonetheless, we were able to measure near-fault velocity fields that may reveal shallow fault processes.

## Results

**Fig 2:**
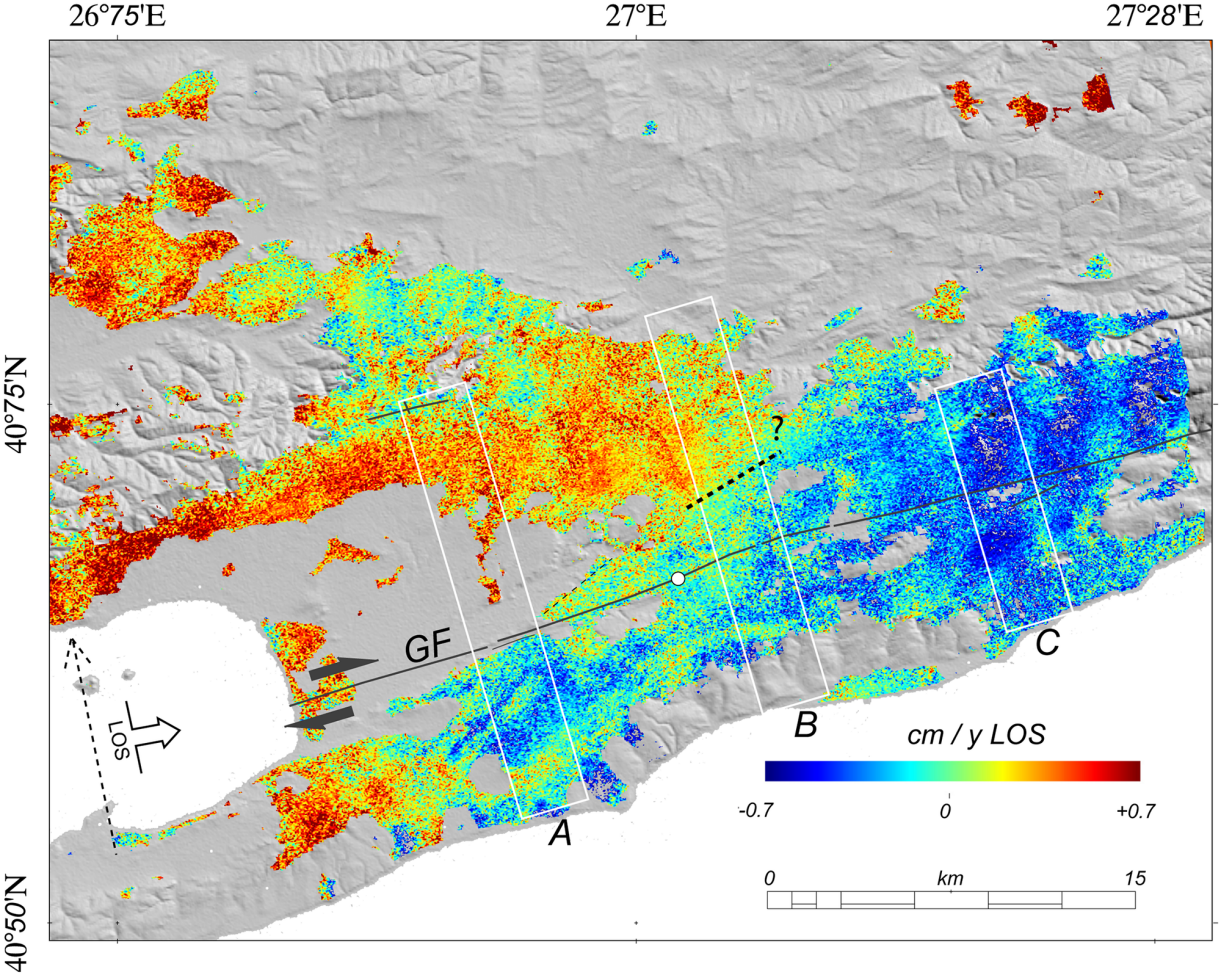
InSAR velocity map. The L-band InSAR velocity map that resulted from stacking 39 interferograms and showing LOS and flight directions. The white boxes on this map mark the source areas used for profile stacking A, B, C (Fig 3). The continuous black line represents the surface trace of the GF, black arrows indicate the sense of motion of the GF. Dotted black segments with the question mark represent possible active faults in the study area. The white circle represents the reference area for the stacking procedure.

**Fig 3:**
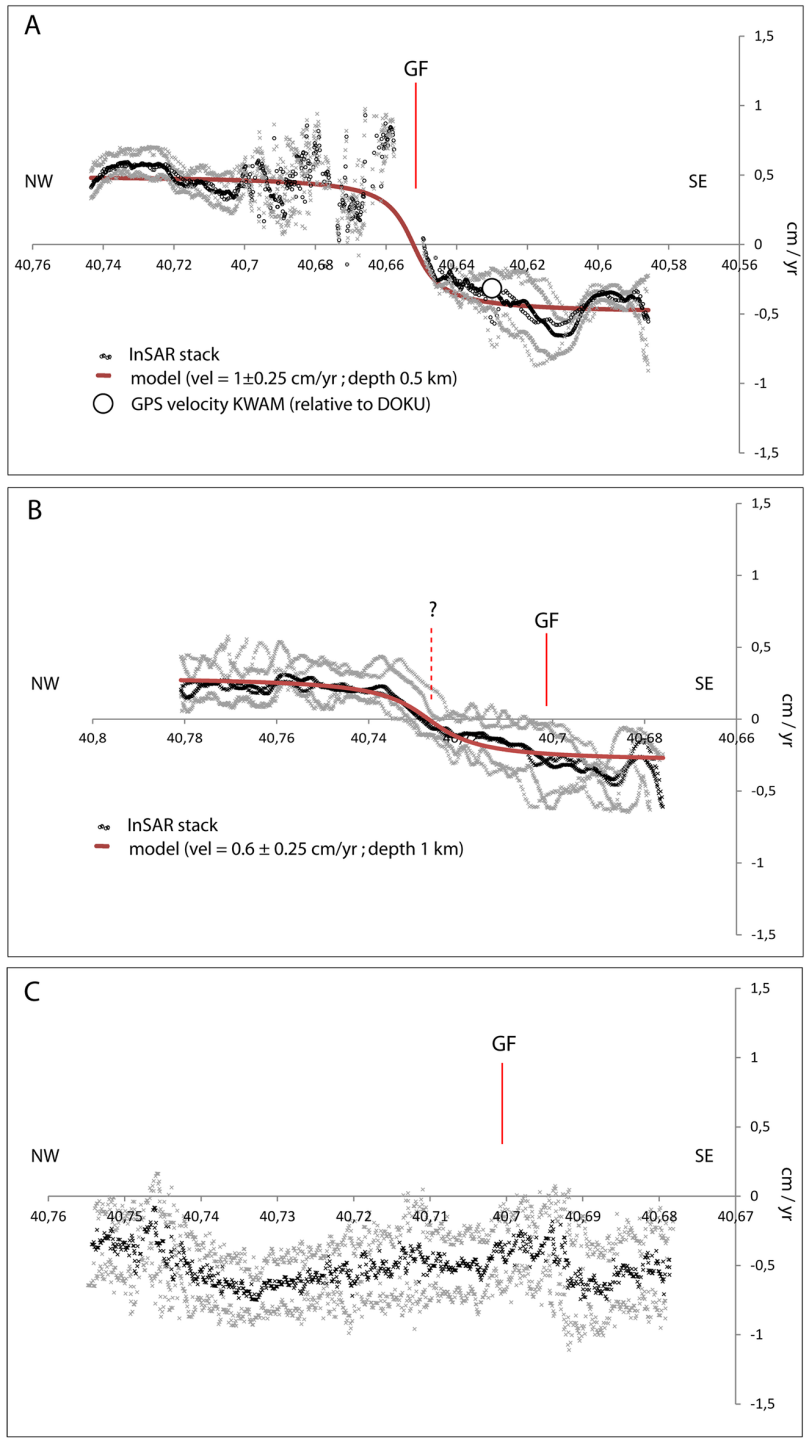
Cross GF velocity profiles. Surface displacement measured on the basis of the stacked profiles within boxes in Figs 1 and 2 (see also S1 and S2 Tables). The black and gray dots on this figure denote InSAR mean values and standard deviations, respectively, while the dark-red line denotes our elastic dislocation 2D model, and the white circle represents GPS velocity measured at the KAVAK station over the same period (Ergintav et al., 2014). The standard deviation of the measurements profiles is calculated as ±0.24 cm/yr (profile A), ±0.21 cm/yr (profile B), ±0.25 cm/yr (profile C).

The first outcome of this study is a SAR phase stack measured in the LOS direction of the sensor encompassing the period between 2007 and 2011 (Fig 2) and resulting in a velocity map. These results show that the SAR signal is consistent over the study area, ca. 2/5 of the full PALSAR frame (S1 Fig). A clear bimodal distribution of surface displacement is present, consistent with dextral shear. We identified three sections of the GF area (Fig 2), each of which shows a characteristic behavior, and traced three cross-fault profiles across them to better visualize surface displacement (Figs 1 and 2). Quite a few areas in the scene (including the area close to section A in Fig 1) are affected by low signal coherence due to temporal surface changes related to agricultural activities. Thus, we illustrated displacement values by stacking a number of cross-fault profiles (Fig 3). The results of this approach show that in section A, the westernmost part of the inland GF, there is clear evidence for near field displacement, possibly due to the shallow motion of the fault on a smoother patch on the fault plane. At the same time, in section B, the middle segment of the GF, evidence for transitional displacement is present, including a smoother gradient from north-to-south. This movement is due either to deepening of the motion of this fault, or previously undetected shallow motion of a fault branch related to the Ganos mountains (the question mark in Fig 2). Finally, results show that there is no measurable surface motion on the easternmost inland section of the GF in section C, which corroborates the hypothesis of a deep fault locking, as proposed in previous work (e.g. [15]). We emphasize that these fault motion interpretations do correspond with features inferred on the basis of regional tectonics but which cannot be identified with our InSAR dataset.

Thus, using two-dimensional (2D) modeling, we attempted to address the question, if our results suggest shallow fault patch motion on multiple section of the GF zone, then how shallow is this smooth patch responsible for motion and how does it develop spatially? To do this, we applied the equations suggested by Smith-Konter et al. [32] to compare our results over three stacked profiles. However, as our data do not include regional tectonics, our fault model cannot be propagated conceptually to an infinite depth but just to the upper tip of a shallow smooth patch. We know that the locking depth of the GF in this area has already been established by GPS at ca. 8 km and that it has been inferred to have a ca. 2 cm/yr velocity (e.g. [15]). On this basis, we attempted to characterize the depth of the shallow smoother patch that could potentially be responsible for the displacement signal measured at the surface in the near field of the GF. An elastic dislocation model can be used to describe the accumulation of elastic strain along a simplified vertical strike-slip fault, while a velocity profile modeled across the fault zone is given by the following equation:

$$v\left(x\right)=\frac{V}{\pi }tan^{-1}(\frac{x}{D}).$$ (1)

In this expression, V denotes the far-field velocity, *x* is the horizontal fault-perpendicular distance, and D is the depth of the upper tip of the smooth shallow patch. However, because our InSAR results do not encompass regional displacement, we did not use Eq (1) to determine locking depth but rather the depth at which the shallow smooth patch is creeping, and its velocity on three selected GF sections (Fig 2). The best-fit interpretation of these data is shown in Fig 3, suggesting shallow segmentation of the GF. Our observations fall within a certain level of incertitude; the standard deviation of the measurements profiles is calculated as ±0.24 cm/yr (profile A, Fig 3), ±0.21 cm/yr (profile B, Fig 3), ±0.25 cm/yr (profile C, Fig 3). The profile model presented in Fig 3a is compatible with motion on a localized smooth patch of the GF at a depth of at least 0.5 km, creeping locally at ca. 1±0.25 cm/yr, while the model profile in Fig 3b suggests that the shallow smooth patch on the GF is no longer present. In this model, shallow motion on a localized smooth patch may be present on a different branch of the GF zone, while the profile in Fig 3c suggests no shallow motion on this section of the GF, as has been previously proposed (e.g. [15]).

## Discussion and conclusions

We utilized L-band InSAR stacking in this study to map surface displacement in the near field of the GF between 2007 and 2011. There were two fundamental motivations for this research project, the first of which is the need to create a spatially detailed map of crustal displacement in the GF section of the NAF. Because this section is seismogenic, it is important to understand how tectonic strain is partitioned across the region, while the second motivation for this project is the fact that the L-band SAR signal is less affected by vegetation cover than its C band counterpart. Therefore, the signal coherence of L-band InSAR is higher across the study area which enables us to retrieve a spatially detailed surface displacement map. At the same time, however processing this particular dataset is challenging, for three reasons. In the first place, the dataset archive is not densely populated, and secondly comprises FBS and FBD data, which need to be coherently combined to increase dataset population. Thirdly, SAR data for this region are heavily affected by RFI which needs to be carefully removed. Thus, to increase the size of our dataset, we combined HH FBS and HH-HV FBD ALOS PALSAR data together, while during our focusing procedure, we carefully filtered RFI. Because the SAR phase stack we calculated for the period between 2007 and 2011 (Fig 2) is coherent over a wide area in the near field of the GF, our results complement previous interpretations for the mechanics of this area suggested by GPS data and suggest that creep on the GF was ca. 2 cm/yr to a locking depth of ca. 8 km. Although our processing removes a phase ramp from the final stack and long wavelength tectonic surface motion driven by fault movement at depth cannot be measured, we are able to measure near-fault velocity fields based on this approach that might reveal shallow fault processes occurring above those at depth. Indeed, our results suggest that the GF is characterized by along strike variations in near field surface motion, possibly due to the motion of shallower faults that together sum to equal the deeper structure hypothesized by Ergintav et al. (2014). Although our results for profile 3a suggests that this section of the GF is characterized by the motion of a shallow smoother patch on the fault plane, the question of whether, or not, this is driven by lithological contrast in the area as soft quaternary sediments are juxtaposed with middle-upper Eocene turbidites remains unanswered. We interpret this localized deformation as a linear-with-time trend just because of the temporally sparse data sampling. Alternatively, we could be observing a transient episodic slip reaching the surface, as recently observed elsewhere on a different section of the NAFZ [18]. Our modeling approach suggests that the fault motion detected in profile 3b may occur on a segment parallel to the GF, as the dotted line (the question mark) in Fig 3 falls within the Eocene turbidite pro-delta lithology, and appears to be orientated parallel with anticlinal fold axes in the region [7]. While this preliminary interpretation will require further verification, profile 3c nevertheless suggests that there is no evidence for surface creep along this section of the GF. This might provide further evidence that tectonic strain is accumulating in this region, which has important implications for seismic cycle and potential, as this section of the GF last ruptured in 1912. Further analysis will be required to fully determine how strain partitioning in this region influences the interactions between segments as well as the seismic potential of the area. Such an analysis might be carried out via improvements of the GPS network in the area, by exploiting next generation L-band SAR data from the ALOS-2 PALSAR sensor, and by combining these data with the high temporal revisit capability of C-band SAR onboard the Sentinel-1 mission.

## Supporting information

S1 Fig Example interferograms on the study region. From the 39 coherent ALOS PALSAR interferograms used in this study, we show examples of four representative interferograms spanning 690 days, 644 days, 322 days and 92 days. The full PALSAR frame is shown. The red rectangle represents the common area covering the GF (Figs 1 and 2) where the SAR signal is coherent over the observation time (2007–2011). The interferometric phase is unwrapped; the color scale represents the interferometric phase modulation between 0–2π (corresponding to 0–11.75 cm LOS).
(TIF)

S1 Table Raw data for cross GF velocity profiles. Surface displacement measured on the basis of the stacked profiles within boxe “A” in Fig 2.
(XLSX)

S2 Table Raw data for cross GF velocity profiles. Surface displacement measured on the basis of the stacked profiles within boxe “B” in Fig 2.
(XLSX)
