# Supplementary figures and images for: An L-band interferometric synthetic aperture radar study on the Ganos section of the north Anatolian fault zone between 2007 and 2011: Evidence for along strike segmentation and creep in a shallow fault patch
Source: PLoS One. 2017 Sep 29;12(9):e0185422. doi: 10.1371/journal.pone.0185422 (PMC5621685; doi:10.1371/journal.pone.0185422)

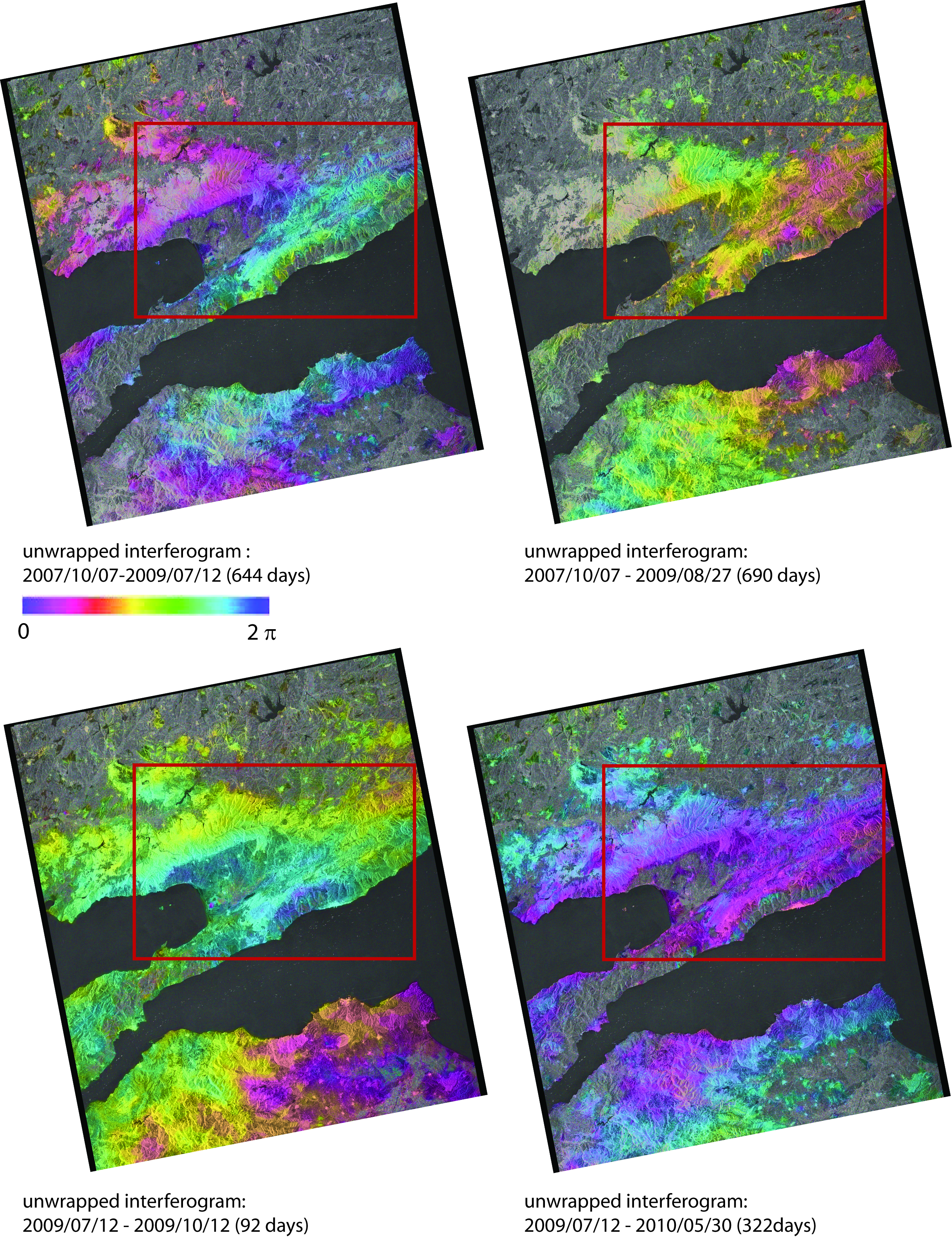

Supplement: S1 Fig — From the 39 coherent ALOS PALSAR interferograms used in this study, we show examples of four representative interferograms spanning 690 days, 644 days, 322 days and 92 days. The full PALSAR frame is shown. The red rectangle represents the common area covering the GF (Figs 1 and 2) where the SAR signal is coherent over the observation time (2007–2011). The interferometric phase is unwrapped; the color scale represents the interferometric phase modulation between 0–2π (corresponding to 0–11.75 cm LOS). (TIF) [file pone.0185422.s001.tif]
